# An empirical test of the bet‐hedging polyandry hypothesis: Female red flour beetles avoid extinction via multiple mating

**DOI:** 10.1002/ece3.7418

**Published:** 2021-03-18

**Authors:** Kentarou Matsumura, Takahisa Miyatake, Yukio Yasui

**Affiliations:** ^1^ Laboratory of Entomology Faculty of Agriculture Kagawa University Kagawa Japan; ^2^ Laboratory of Evolutionary Ecology Graduate School of Environmental and Life Science Okayama University Okayama Japan

**Keywords:** bet‐hedging, extinction avoidance, monandry, polyandry, risk spreading, *Tribolium castaneum*

## Abstract

Bet‐hedging via polyandry (spreading the extinction risk of the female's lineage over multiple males) may explain the evolution of female multiple mating, which is found in a wide range of animal and plant taxa. This hypothesis posits that females can increase their fitness via polyandrous mating when “unsuitable” males (i.e., males causing reproductive failure for various reasons) are frequent in the population and females cannot discriminate such unsuitable mates. Although recent theoretical studies have shown that polyandry can operate as a bet‐hedging strategy, empirical tests are scarce. In the present study, we tested the bet‐hedging polyandry hypothesis by using the red flour beetle *Tribolium castaneum*. We compared female reproductive success between monandry and polyandry treatments when females mated with males randomly collected from an experimental population, including 20% irradiated (infertile) males. In addition, we evaluated geometric mean fitness across multiple generations as the index of adaptability of bet‐hedging traits. Polyandrous females showed a significantly higher egg hatching rate and higher geometric mean fitness than monandrous females. These results strongly support the bet‐hedging polyandry hypothesis.

## Graphical abstract

We compared the fitness between monandrous females and polyandrous females when unsuitable male that females could not be identified was included in the population using the red flour beetle (*Tribolium castaneum*). The results showed that polyandrous females increased the fitness than monandrous females. These results provide clear evidence that the bet‐hedging hypothesis can explain the evolution of female multiple mating.

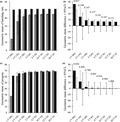

## 1 INTRODUCTION

In many animals, male fitness is positively related to the number of mates that he gets because males produce an enormous number of sperm and potentially fertilize all eggs of partners (Bateman, 1948), whereas female fitness does not monotonically increase in response to multimale mating because the limited egg production of females determines the upper limit of fitness (Bateman, 1948). Moreover, multiple mating is usually costly and risky for females (e.g., time and energy consumption and increased predation and infection; Arnqvist & Rowe, 2005; Harano et al., 2006). Nevertheless, female multiple mating is ubiquitous in many animals (and plants); thus, the evolutionary significance of polyandry has received considerable attention from many evolutionary biologists (e.g., Jennions & Petrie, 2000; Meade et al., 2017; Nason & Kelly, 2020; Pizzari & Wedell, 2013; Simmons, 2005; Yasui, 1998; Zeh & Zeh, 2003).

To explain the evolution of female multiple mating, various hypotheses have been proposed. For example, if females receive direct benefits (e.g., replenishment of the sperm supply, nutrients in the seminal fluid, protection against predators, and paternal care of offspring) from males in exchange for copulation, multiple mating may be adaptive for the females (Arnqvist & Nilsson, 2000; Yasui, 1998). In the absence of direct benefits, if females obtain some genetic (indirect) benefits for the offspring (e.g., good genes or genetic diversity) from males, then polyandrous females would be favored (Jennions & Petrie, 2000; Yasui, 1998). These hypotheses have been investigated by numerous empirical and theoretical studies (e.g., Jennions & Petrie, 2000; Meade et al., 2017; Nason & Kelly, 2020; Pizzari & Wedell, 2013; Simmons, 2005; Zeh & Zeh, 2003). Today, the increasing studies have reported the benefits of female multiple mating with different males (e.g., García‐González et al., 2015; Lewis et al., 2020; Power & Holman, 2014; Snook, 2014). However, it is still controversial whether the costs of remating could be compensated by the proposed benefits.

Many genetic‐benefit hypotheses implicitly presuppose that females cannot discriminate males according to their quality before mating. However, they also assume that females can employ some mechanisms to bias paternity toward particular males after multiple matings (Yasui, 1998). Because if precopulatory discrimination is practicable and reliable, females should mate with only the best male among the potential mates (i.e., monandry). In terms of postcopulatory mechanisms, the good‐sperm hypothesis (Yasui, 1997) assumes that females cannot detect males possessing good genes before mating but that sperm competition chooses the good‐gene male after multimale matings because sperm competition ability is positively correlated with genetic quality of males (Yasui 1997). Here, sperm competition functions as a process of “indirect mate choice,” through which females acquire high‐quality males simply by accepting the winner of the male–male competition (Wiley and Poston, 1997; Saether et al., 2005).

However, reliable mate choice is often difficult, even after multiple matings. In fluctuating environments, the fittest genotypes change between generations (Yasui, 1998, Yasui & Garcia‐Gonzalez, 2016). Even in stable environments, low‐quality males may conceal their own quality (sexual conflict; Arnqvist & Rowe, 2005). Various temporary or permanent genetic or environmental factors cause male infertility (Garcia‐Gonzalez, 2004; Hasson and Stone, 2009; Rhainds, 2010; Tyler & Tregenza, 2013; Forbes, 2014; Greenway et al., 2015; Greenway and Shuker, 2015). For example, some postemergence injuries or infectious diseases may damage male copulatory organs, and males may temporally exhaust their sperm stock. Males may bear new deleterious mutations or possess genetic elements that are incompatible with the female genes. These factors recurrently generate a considerably high frequency of unsuitable males in every population (Garcia‐Gonzalez, 2004). A previous study reported that the proportion of infertile matings across 30 insect species is surprisingly higher than the previously thought, varying between 0% and 63%, with a median of 22% (Garcia‐Gonzalez, 2004).

If females cannot discriminate unsuitable males, monandrous mating with such males will be lethal to the own lineage (Yasui & Garcia‐Gonzalez, 2016; Yasui & Yoshimura, 2018). However, indiscriminative polyandrous mating could allow females to avoid this problem. The logic is very simple; if the unsuitable male frequency in a population is 0.2, then monandry is expected to fail with a probability of 0.2, but in the case of two‐male (*n*‐male in general) polyandry, this value decreases to only 0.04 (0.2*^n^*). This idea is known as the “bet‐hedging polyandry” hypothesis (Yasui, 1998, 2001; Yasui & Garcia‐Gonzalez, 2016; Yasui & Yoshimura, 2018). In life‐history evolutionary theories, bet‐hedging means the adaptation of extinction avoidance in unpredictably fluctuating environments (Philippi & Seger, 1989; Slatkin, 1974). In the changing environment (different mates in our context), fitness necessarily varies among individuals of the same strategy (genotype). Some females mate with suitable males and achieve high fitness but others mate with unsuitable males and result in low fitness. To evaluate such varying individual fitness, mean fitness among individuals of the same strategy is calculated. To average fitness scores within a generation, the arithmetic mean (*W*_WG_) of all individuals of the same strategy should be used, while the between‐generation mean fitness (*W*_BG_) should be the geometric mean of the *W*_WG_ across multiple generations (Philippi & Seger, 1989; Slatkin, 1974; Yasui, 1998, 2001; Yasui & Garcia‐Gonzalez, 2016). As a general term, bet‐hedging is the strategy that sustains higher geometric mean fitness over generations and avoids the extinction of the genotype controlling this strategy.

Some theoretical studies have investigated whether polyandry works as bet‐hedging (e.g., Yasui, 1998, 2001; Yasui & Garcia‐Gonzalez, 2016; Yasui & Yoshimura, 2018). Simulations by Yasui and Garcia‐Gonzalez (2016) show that if ca. 22% of the males in a population are infertile (according to the estimate of Garcia‐Gonzalez, 2004) and females cannot discriminate such males, polyandry achieve higher fixation probability than monandry in the structured metapopulations.

However, limited empirical studies have been performed to test this hypothesis (but see Fox & Rauter, 2003; Garcia‐Gonzalez et al., 2015; Lewis et al., 2020; Power & Holman, 2014; Schmoll et al., 2007; Yuta et al., 2018). In particular, very few experimental studies examined whether polyandry enhances the geometric mean fitness across successive generations (Fox & Rauter, 2003; Garcia‐Gonzalez et al., 2015).

In this study, we tested the bet‐hedging polyandry hypothesis by using the red flour beetle *Tribolium castaneum*. *T. castaneum* is highly promiscuous insect throughout their adult lives (Pai et al., 2007; Sokoloff, 1974), and many studies have used this species as the model system of female multiple mating (e.g., Bernasconi & Keller, 2001; Pai et al., 2005, 2007; Pai & Yan, 2002, 2003). We controlled the unsuitable male frequency in the experimental population by using Co‐60 gamma‐ray irradiation. We compared reproductive success between monandrous and polyandrous females when an unignorable frequency of irradiated males existed in the population. In addition, we evaluated the geometric mean fitness of females employing the two mating strategies across multiple (simulated) generations. Our predictions are as follows: monandrous females produce either fertile clutch (almost all eggs are fertile) or infertile clutch (almost all eggs are infertile) but polyandrous females leave partially infertile clutch (some eggs are infertile but others are successful within the same clutch). Consequently, the interfemale variance in fitness (egg hatching rate) is greater in monandrous treatment than polyandrous treatment. If we consider the females in the same treatment as the successive generations of a single lineage adopting each strategy (i.e., simulation using real data), the geometric mean fitness across “generations” should be greater in polyandry than monandry because of the smaller fluctuation of fitness across generations in polyandry.

## 2 MATERIALS AND METHODS

### 2.1 Insects and culture

The laboratory population of *T. castaneum* used in this study has been maintained for more than 40 years according to the method described in Suzuki and Nakakita (1991). The beetles were cultured in incubators (Sanyo, Japan) maintained at 25°C with a 16 L:8 D (7:00 lights on, 23:00 lights off) light cycle. The beetles were fed whole meal flour (Nisshin Seifun, Japan) including beer yeast (Asahi beer, Japan). More details of the culture methods are described in Miyatake et al. (2004).

The sterile male technique is a common method to evaluate sperm competition, widely adopted since Parker (1970). Appropriate sublethal doses of irradiation induce male sterility but do not affect male courtship behavior (e.g., Magris et al., 2015; Schneider et al., 2006). We used irradiated males as unsuitable males. Radiation treatment of *T. castaneum* males was conducted at the Okinawa Prefectural Plant Protection Center (Okinawa, Japan). Virgin males (*n* = 100, 21–28 days old) were randomly collected from the laboratory population, and these males were irradiated with a Co‐60 gamma source at 80 Gy. Although this radiation treatment can substantially reduce male fertility, the probability of the males becoming completely sterile was relatively low (approximately 13.3%; unpublished data). However, radiation stronger than 80 Gy causes reduced longevity of the beetle (personal observation) and may also affect male behavior. If females can discriminate such abnormal males before copulation, the prerequisite of the bet‐hedging polyandry hypothesis is not satisfied. Therefore, we adopted an 80 Gy dose in this study.

### 2.2 Mating experiment

**FIGURE 1:**
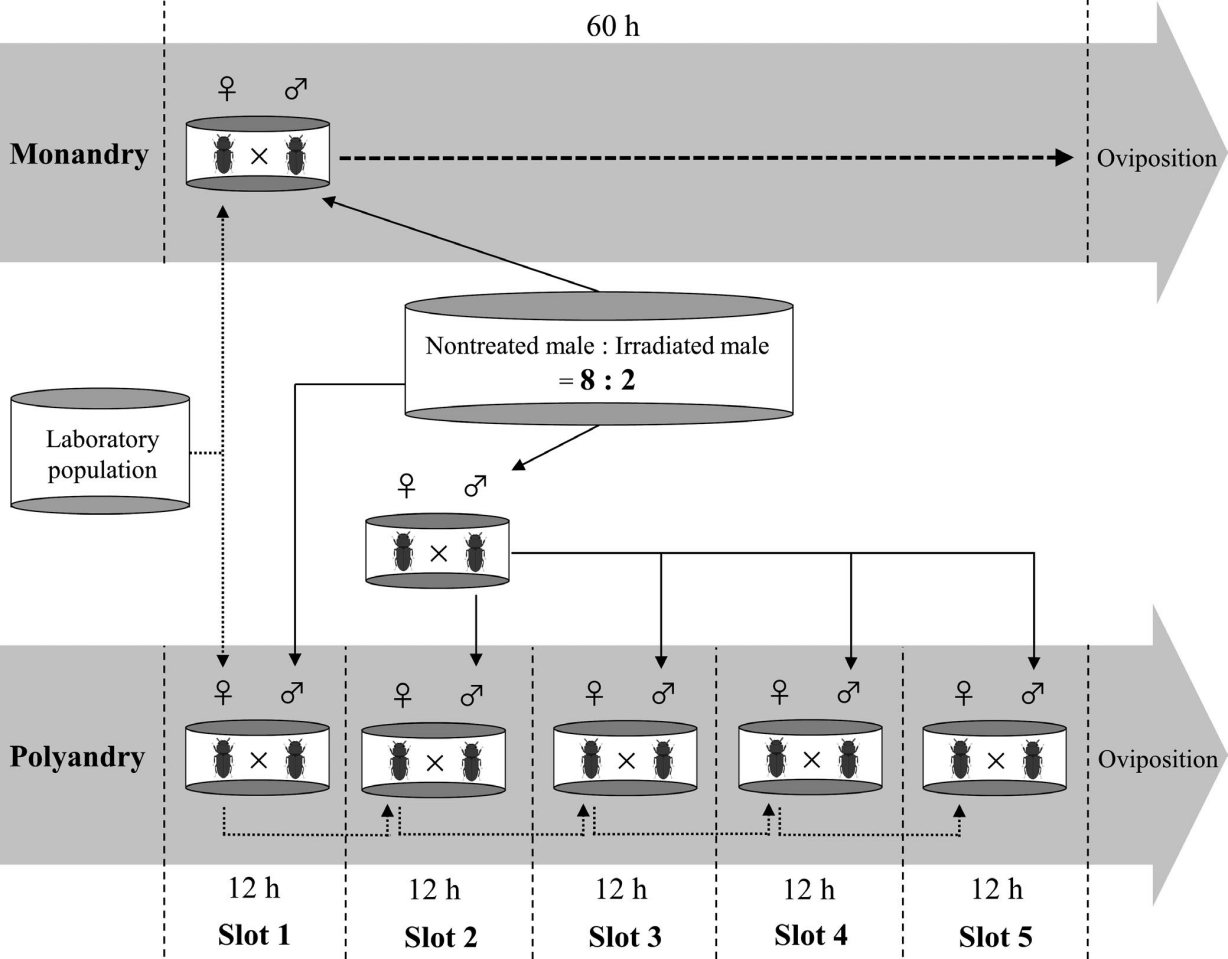
Experimental design involving the monandry and polyandry treatments. In the monandry treatment, a pair was allowed to mate for 60 hr. In the polyandry treatment, a female was paired with a male that was randomly selected from the population including intact and irradiated males for 12 hr (mating slot 1). Subsequently, the female was also paired with four different males, each for 12 hr (slots 2–5; i.e., a female was given opportunities to mate with five males for 60 hr in total). To minimize the difference in mating experience of males between the polyandry and monandry treatments, the second to fifth males in the polyandry treatment (slot 2 to 5) were paired with another female until the experiment (see text)

The experimental design is described in Figure 1. We created an artificial male population that consisted of virgin males from the laboratory population (*n* = 144; “Nontreated male” in Figure 1) and virgin males from the irradiated population (*n* = 36; “Irradiated male” in Figure 1) after sexing in the pupal stage. Thus, the frequency of irradiated males was 20%. This value was chosen to reflect the median frequency of infertile mating in nature (approximately 22%) according to the review of Garcia‐Gonzalez (2004), the simulation study of Yasui and Garcia‐Gonzalez (2016) and an analytical model by Yasui and Yoshimura (2018). Because there is no visual difference between intact and irradiated males, observers (and perhaps females) cannot discriminate them. To distinguish sexes, all males were painted by a white marker (PX‐21, Mitsubishi, Japan) on the elytra. This treatment does not affect the mating behavior of *T. castaneum* (Matsumura & Miyatake, 2015).

Virgin females (*n* = 60, 21–28 days old) were randomly collected from the laboratory population, and each female was put into a Petri dish (diameter 30 mm, height 15 mm) with food. We included 30 females in the monandry treatment and 30 females in the polyandry treatment. A male was randomly collected from the artificial male population and put into the Petri dish for pairing with a female. Note that we sampled only one male per monandrous female but 5 different males per polyandrous female. Thus, the unsuitable male frequency in the samples was expected to be 20% on average among females in both treatments, but the variance (random fluctuation) around the mean was larger in the monandry treatment because the sample size was 1/5 of that in the polyandry treatment (i.e., the law of large numbers). This unpredictable fluctuation of male quality may produce higher geometric mean fitness in polyandrous females than in monandrous females if the sample size is sufficiently small (for the rationale, see Yasui & Garcia‐Gonzalez, 2016). Expecting this random effect, we did not precisely set the male ratio in the samples (such as 6 irradiated/24 intact males in monandry and 30 irradiated/120 intact males in polyandry). The realized ratios are unknown.

In the monandry treatment, a female was allowed to copulate with a male for 60 hr. In the polyandry treatment, a female was allowed to copulate with a male for 12 hr. After 12 hr, the male was replaced with another male from the artificial male population, and the focal female was paired with the new male for 12 hr. This procedure was replicated five times (slots 1–5; i.e., the females in the polyandry treatment were paired with 5 males for 12 hr each and 60 hr in total). Because the small body size of *T. castaneum* makes it difficult to confirm its mating success (whether the male's genitalia were coupled with the female's genitalia), we did not record the number of matings in each treatment. Although *T. castaneum* females are extremely promiscuous (maximum of 12 copulations in one hour; Pai & Yan, 2003, Pai et al., 2007), males often fail to correctly insert the genitalia or transfer sperm (with a maximum probability of 55%; Tyler & Tregenza, 2013). Thus, even if the number of matings was recorded, the data might include such mating failures. Instead, we adopted a method in which each pair was allowed to copulate freely for 12 hr (in the polyandry treatment) or 60 hr (in the monandry treatment). With this procedure, we cannot completely exclude the possibility that females in the polyandry treatment mated monandrously with a single suitable or unsuitable male. However, considering the high mating frequency in this species, it is highly unlikely that the females confined to a small space with a male did not mate for 48 hr. If this were the case, the fitness variance among “polyandrous” females would increase to the same level observed in the monandry treatment (because the mating would be equivalent to monandry), and the bet‐hedging effect (geometric mean fitness) would be diminished. Therefore, we conservatively evaluated the fitness of the polyandrous females and tried to detect their advantage over monandrous females.

During the 60‐hr pairing in the monandry treatment, the male condition (e.g., the propensity for mating) might have changed (he might have tired). If only virgin males were used in the polyandry treatment, the difference in male condition between treatments would be great in the late period of the 60‐hr pairing. To avoid this, the males in the polyandry treatment were confined with a different female before being paired with the focal female. For example, the male in slot 4 (Figure 1) was isolated with a nonfocal female during the 36 hr prior to his turn. Therefore, the condition of males (i.e., the number of matings that they experienced) was adjusted between the monandry and polyandry treatments.

After 60 hr of pairing, each female was allowed to oviposit for 2 weeks, and the numbers of eggs and newly hatched larvae were recorded (Figure 1). Because one polyandrous female did not lay eggs, we omitted this female's data from statistical analysis (i.e., monandrous female: *n* = 30, polyandrous female: *n* = 29). All experiments were conducted in the laboratory at 25°C.

### 2.3 Statistical analysis

To analyze the numbers of eggs and larvae, and hatching rate, we used a generalized linear model (GLM). AIC value was used to select an appropriate statistical model for the tests of number of eggs and larvae (Table S1). Because their AIC values were the smallest, the gamma and Gaussian distributions were adopted in the tests of the number of eggs and larvae, respectively (Table S1). Because the hatching rate (larvae/eggs) is binary data (hatched or unhatched), we used a GLM with a binomial distribution. These analyses were conducted in R version 3.4.3 (R Core Team, 2017).

In addition, we compared geometric mean fitness within a simulation framework using obtained data (i.e., real data simulation). The remarkable feature of geometric mean is that it is sensitive to small value, especially 0 in the samples. If only one female with a hatching rate of zero exists in the sample, the geometric mean necessarily becomes zero (e.g., $\sqrt[{4}]{1\times 1\times 1\times 0}=0$, meaning the extinction at the 4th generation). Because we wished to compare the likelihood of reproductive failure (the probability of zero fitness) between treatments, zero‐hatching data were replaced with 0.000001 to calculate geometric mean fitness. We used PopTools version 3.2.5 (Hood, 2011) for resampling and randomization tests comparing the geometric mean fitness between treatments. The 30 monandrous and 29 polyandrous females were randomly divided into 1–30 virtual generation(s). There were 8 possible combinations of the number of females per generation and the (simulated) successive number of generations (Table 1).

**TABLE 1:** Division pattern of *n* females into *G* generation(s) in the randomization test comparing monandry and polyandry

Comparison	Monandry (*n* = 30)	Polyandry^a^ (*n* = 29)
1	1♀30*G*	1♀29*G*
2	2♀15*G*	2♀14*G* + 1♀1*G*
3	3♀10*G*	3♀9*G* + 2♀1*G*
4	5♀6*G*	5♀5*G* + 4♀1*G*
5	6♀5*G*	6♀4*G* + 5♀1*G*
6	10♀3*G*	10♀2*G* + 9♀1*G*
7	15♀2*G*	15♀1*G* + 14♀1*G*
8	30♀1*G*	29♀1*G*

^a^ In the comparison 2–7, one generation includes one less female than other generations because of *n* = 29.

For example, in the comparison 4, 30 samples in monandry were divided into 6 generations each including 5 females and 29 samples in polyandry were divided into 5 generations each including 5 females and 1 generation including 4 females. We calculated arithmetic mean among 5 or 4 females regarding each fitness parameter (e.g., egg hatching rate) in each generation. Next, geometric mean of the arithmetic means was calculated across 6 virtual generations of the same treatment. The difference of the geometric mean fitness (polyandry–monandry) was used as the test statistic. We iterated this procedure 100,000 times and obtained the mean and variance of the test statistic. To calculate observed test statistic, the shuffling was carried out within each treatment (*n* = 30 and 29), while for null‐hypothesis test statistic, it was conducted over pooled samples (*n* = 59). From the extent of overlap between the distributions of observed and null‐hypothesis statistics, the significance level (P) was estimated.

## 3 RESULTS

**FIGURE 2:**
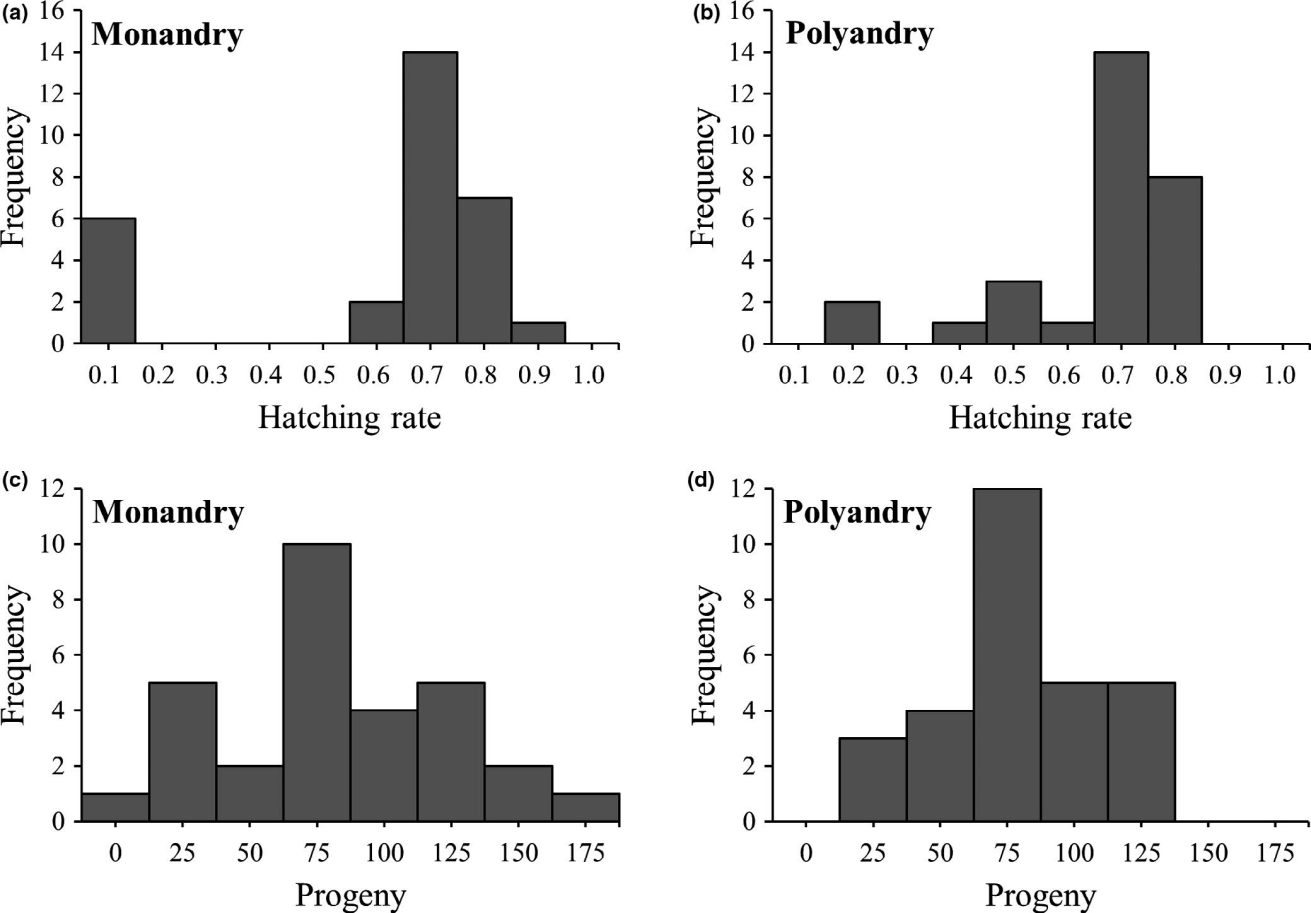
Frequency distributions of the egg hatching rate of the monandrous (*a*) and polyandrous (*b*) females and the number of larvae of monandrous (*c*) and polyandrous (*d*) females

Out of the 59 total experimental females, 6 females assigned to the monandry treatment showed an egg hatching rate less than 0.1. For these females, the numbers of larvae/eggs were 0/127, 2/86, 4/81, 5/68, 7/144, and 9/127, respectively. Because we used a gamma‐ray dose that induces less than 100% sterility to avoid influences on male behavior, we considered these females to copulate with only unsuitable males. Thus, we categorized a hatching rate less than 0.1 as complete reproductive failure (as mentioned, these data were replaced with 0.000001 only at the geometric mean analysis, not at the other analyses such as arithmetic mean). The egg hatching rate in the monandry treatment showed a bimodal distribution, including highly successful (>0.9) and totally failed (<0.1) females (Figure 2a), whereas the distribution in the polyandry treatment did not include such extremes (Figure 2b). Similar distributions were observed for the number of larvae (Figure 2c,d).

**FIGURE 3:**
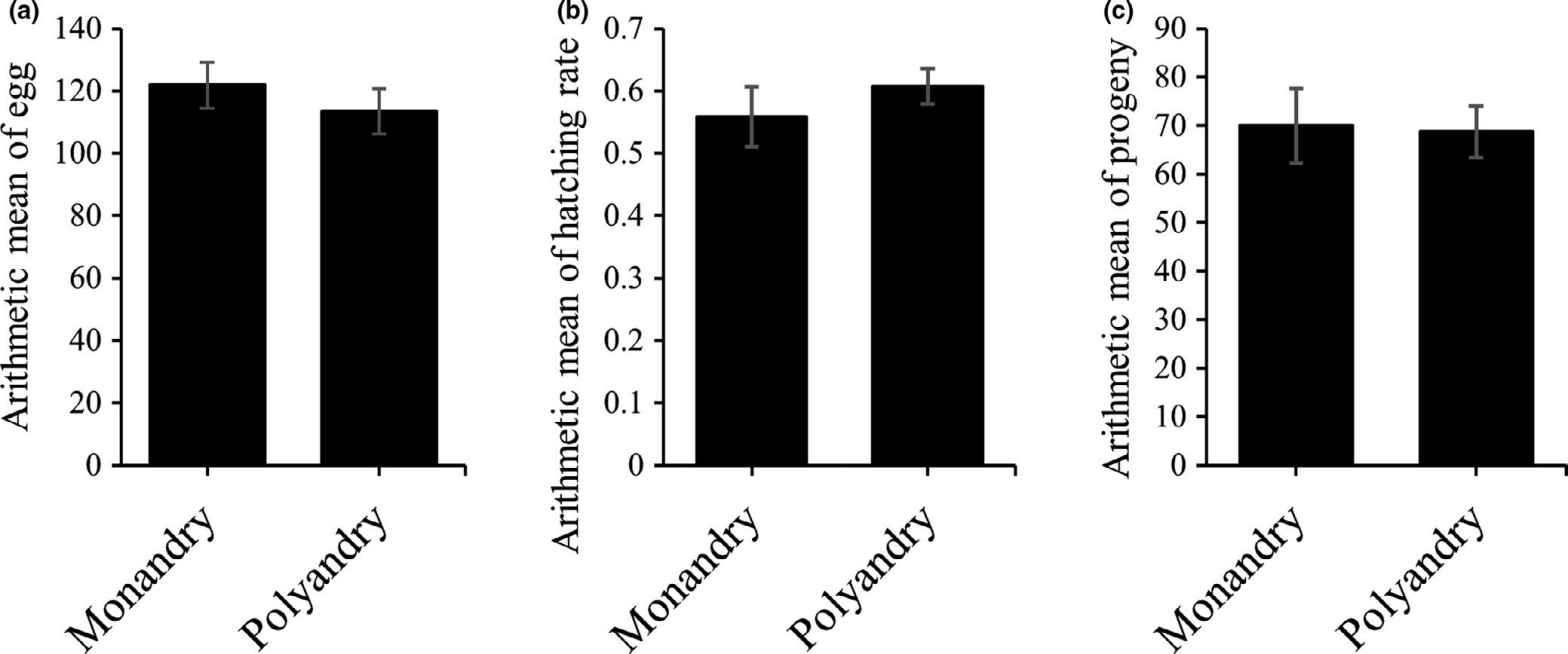
Comparison of (*a*) the arithmetic mean of the number of eggs, (*b*) the egg hatching rate, and (*c*) the number of larvae between the monandry and polyandry treatments. Error bars show the standard error (SE)

Figure 3abc shows the arithmetic means of the number of eggs, egg hatching rate, and number of larvae, respectively (*n* = 30 in monandrous treatment, *n* = 29 in polyandrous treatment). Although there was no significant difference in the number of eggs between treatment (GLM; *χ* ^2^_1,57_ = 0.66, *p* =.42; Figure 3a), polyandry had a significantly higher hatching rate than monandry (GLM; $\chi_{1,57}^{2}$ = 7.14, *p* =.0075; Figure 3b). There was no significant difference in the number of larvae between treatments (GLM;  = 0.02, *p* =.89; Figure 3c).

**FIGURE 4:**
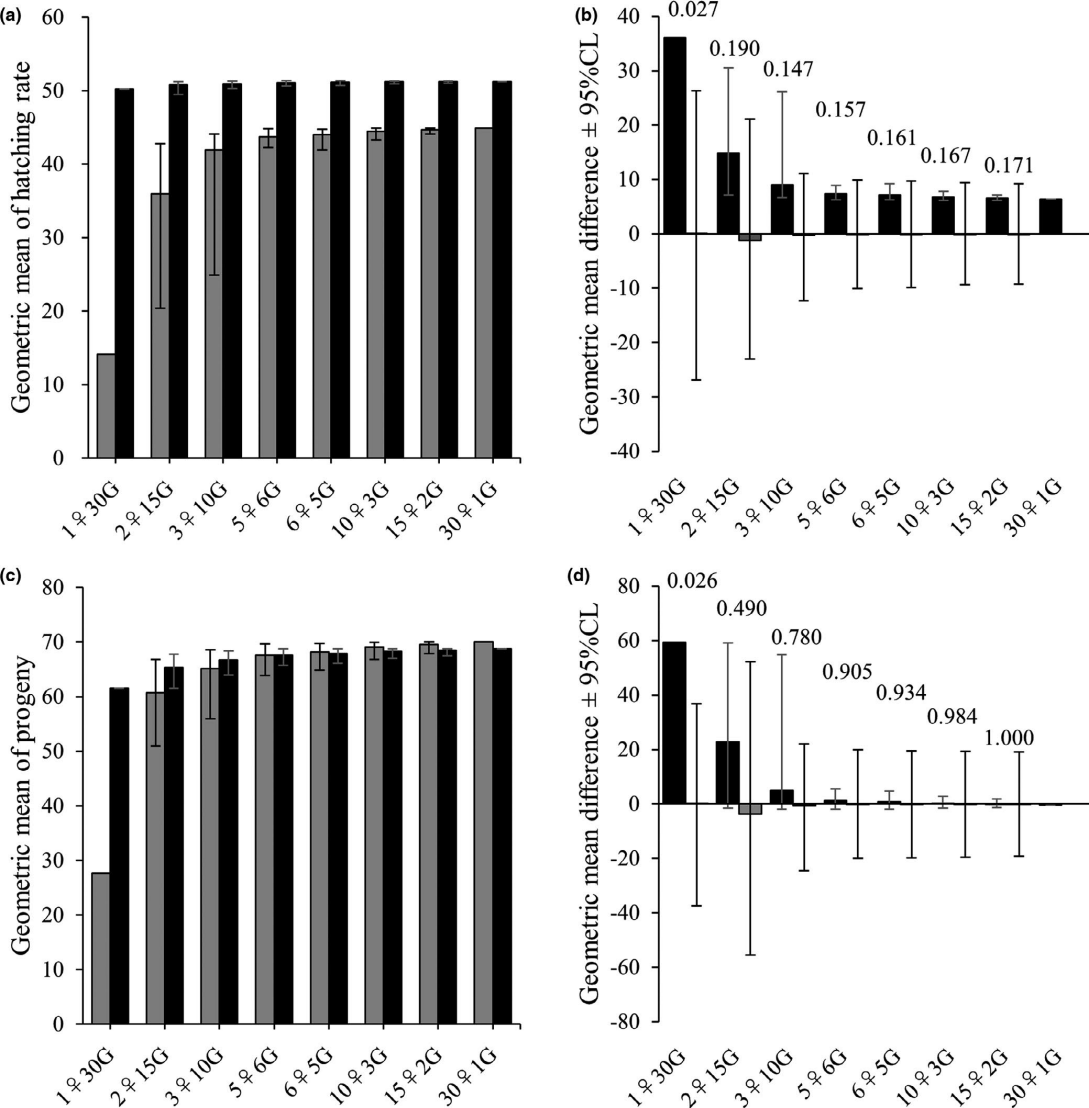
Geometric means of the egg hatching rate (*a*) and number of larvae (*c*) for various division of samples (e.g., 2♀15G means 15 successive generations, each including 2 females) (see Table 1). In polyandry, sample size in a generation is one less than the other generations (not shown in x‐axis). Polyandry (black bar) and monandry (gray bar). Comparison of geometric mean fitness was made across various numbers of virtual (simulated) generations regarding the angular‐transformed egg hatching rate (*b*) and number of larvae (*d*). Values above the bars are *p*‐values (2‐side test). Error bars show the 95% confidence range obtained from 100,000 iterations of shuffling. For 1♀30 or 29G, there was only one combination of sampling, so the observed fitness has no variance. For 30 or 29♀1G, the sample size (no. generations) was one, and thus, no variance exists

Figure 4 shows the geometric means of the egg hatching rate (*a*) and number of larva (*c*) for the 8 combinations of the number of females per generation and the number of generations (Table 1). Polyandrous females showed a significantly higher geometric mean than monandrous females about the egg hatching rate and the number of larva only for the combination 1♀30G (Figure 4bd).

## 4 DISCUSSION

### 4.1 Polyandry works as bet‐hedging

Polyandrous females of *T. castaneum* that were paired with five males exhibited a higher egg hatching rate than monandrous females when irradiated males were included in the male population. Because of the limited gamma‐ray dose, we assumed that the females were unable to discriminate the unsuitable males. Thus, monandrous mating with an unsuitable male would result in total reproductive failure (extinction of female's own lineage). On the other hand, because the probability that all five partners were unsuitable males was extremely low (0.2^5^ = 0.00032), polyandrous females were able to avoid extinction (at least some offspring survived). Furthermore, polyandrous females showed significantly higher geometric mean fitness than monandrous females for the 1♀29 or 30G combination, in which only one female lineage employed the same strategy (polyandry or monandry) across 29 or 30 generations. In such cases, reproductive failure (0 fitness) of only one female in any generation causes extinction of the lineage. Even in this severe situation, polyandry can allow females to avoid extinction by risk‐spreading over multiple males. Interestingly, the differences between polyandry and monandry gradually decreased with an increase in the number of females per generation (see Figure 4). This coincides with the theory that bet‐hedging via polyandry is effective only in small female populations or small subpopulations constituting metapopulations because in large populations, the failure of unlucky monandrous females is offset by the success of lucky monandrous females (Yasui & Garcia‐Gonzalez, 2016). Because the within‐generation mean fitness of a genotype is calculated as the arithmetic mean, monandrous genotypes are unlikely to go extinct if many individuals exist in a generation. In other words, producing more than one offspring effectively functions as risk‐spreading (another type of bet‐hedging), even if females mate monandrously. However, this effect diminishes in small populations (e.g., the 1♀30G combination, meaning that no spare individuals exist). Therefore, bet‐hedging polyandry is quite an effective strategy if the population frequently experiences bottlenecks (for the relevance of bet‐hedging polyandry to the conservation of endangered species, see Yasui & Garcia‐Gonzalez, 2016).

The frequency distributions of the egg hatching rate and the number of larvae were wider for monandry than for polyandry and only monandry included both extremes. Therefore, monandry is a “high‐risk, high‐return” strategy, while polyandry is a “low‐risk, low‐return” strategy, as predicted by bet‐hedging theory (Yasui & Garcia‐Gonzalez, 2016).

### 4.2 Bet‐hedging and sexual selection can work together

Moreover, the observed increase in the egg hatching rate (Figure 3b) also supports the good‐sperm hypothesis (Yasui, 1997). Polyandrous females showed a trend to lay fewer eggs than monandrous females, suggesting some direct costs of polyandry (e.g., a harmful accessory gland substance; Arnqvist & Rowe, 2005; Harano et al., 2006), but this was compensated for by the higher egg hatching rate in polyandrous females. This is understandable because some good‐gene males (s) among the five mates won sperm competition and increased egg viability via sire effects. Because the sperm competition ability of irradiated males is often inferior to that of normal males (Parker, 1970), such postcopulatory sexual selection could work. Thus, polyandrous females benefited from both the high genetic quality of their offspring and the avoidance of extinction. Note that the two hypotheses are not mutually exclusive because even if postcopulatory paternity skew is possible, some uncertainty always exists in any process (e.g., good‐gene males may accidentally fail to inseminate). Bet‐hedging polyandry can work against such uncertainty because it is unlikely that all normal males will fail for stochastic reasons. The male‐caused reproductive failure may be common not only in this species (Tyler & Tregenza, 2013), but also throughout various taxa, and our finding will contribute to explain the evolution of polyandry that found in many animals and plants.

### 4.3 Problems of the sterile male technique

A problem inherent in mating experiments using sterilized males is the difficulty of determining whether an unhatched egg is caused by the nonoccurrence of mating (i.e., virgin females) or egg mortality after fertilization. Females of *T. castaneum* often lay unfertilized eggs without mating (Sokoloff, 1974). In fact, one monandrous female in our experiment laid 127 unhatched eggs only. Because we did not observe copulations during each experiment, this female may have discriminated unsuitable males and avoided copulation. However, because females of *T. castaneum* are highly promiscuous (Pai et al., 2007; Pai & Yan, 2003), it seems very unlikely that the pair confined to the small space did not copulate for 60 hr. Therefore, the female that laid only unhatched eggs most likely mated (probably repeatedly) with a completely sterilized male rather than rejecting the male throughout the experiment. Furthermore, the other 5 monandrous females that were classified as “mated with unsuitable males” showed a very small nonzero (<0.1) hatching rate (Figure 2a). In a different experiment, the same irradiation treatment induced complete sterility in ca. 13.3% of males (unpublished data). Thus, these 5 females must have mated with unsuitable males without discrimination unless they performed parthenogenesis (no evidence of parthenogenesis exists in this species). Hence, 80 Gy Co‐60 gamma‐ray radiation is an appropriate treatment in this and future studies.

Some previous studies revealed direct benefits (fitness increase within the generation: Lewis & Austad, 1994) and indirect benefits (fitness increase in later generations: Bernasconi & Keller, 2001; Pai et al., 2005; Pai & Yan, 2002) of female multiple mating in *T. castaneum*. Our results suggested an additional benefit of female multiple mating, the long‐term sustainability of the female lineage (or genotype).

In this study, we cannot exclude the possibility that females recognized the gamma‐irradiated males and performed pre‐ and/or postcopulatory mate choice against these males. In fact, females of this species perceive male's precopulatory courtship behavior (elytra rubbing with legs) and perform cryptic female choice against artificially manipulated (tarsal ablation) males (Edvardsson & Arnqvist, 2000). However, it is unlikely that such a low dose of gamma ray induced abnormal courtship behavior on the males at least in the short term. Because the females did not discriminate the unsuitable males and mated randomly, 20% of the monandrous mating (corresponds to the frequency of irradiated males) resulted in the reproductive failure (Figure 2a). Therefore, we are confident that gamma irradiation is not a problem in our results.

We consider that the lower hatching rate than 0.1 in the monandrous treatment as the sire of irradiated males and replaced these data with 0.000001 at the calculation of the geometric mean fitness but this measure did not apply to the rate around 0.2 in the polyandrous treatment (Figure 2). The border line seems arbitrary. However, considering the absent of the moderate hatching rate (0.2–0.5) in the monandry treatment and the 80 Gy radiation induces sufficiently low hatching rate (<0.1) (unpublished data), the bar 0.2 in polyandry should not be replaced with 0.000001. We guess that these polyandrous females mated with an irradiated male as the 5th mate because the last male sperm precedence (*P*_2_ = 0.6–0.9: *P*_2_ is the proportion of offspring fathered by the second male in double mating experiment) has been reported in this species (Fedina & Lewis, 2008; Yuhao et al., 2020). This inference is far more plausible than that the females successively mated with 5 insufficiently irradiated males (otherwise higher hatching rate would be recorded). For the simplicity, the bet‐hedging polyandry hypothesis assumes the complete sperm mixing (Yasui, 1998, 2001; Yasui & Garcia‐Gonzalez, 2016), but even if the last male fertilizes more eggs, this does not affect our logic because polyandry leaves at least a few offspring unless the *P*_2_ value is 1 whereas monandrous mating with an infertile male always leads to extinction. Therefore, the advantage of polyandry is evident.

### 4.4 The difficulty to separate bet‐hedging from sexual selection

This study successfully verified the prediction of the bet‐hedging polyandry hypothesis that a frequency of ca. 20% indistinguishable unsuitable males in a population will favor polyandry (Yasui & Garcia‐Gonzalez, 2016; Yasui & Yoshimura, 2018). From the same experimental population including 20% unsuitable males, polyandry sampled 5 times more males than monandry. Thus, the sampling (in blind) error was larger in monandry than polyandry due to the stochasticity, causing the difference in the geometric mean fitness. However, to separate the effects of bet‐hedging from those of sexual selection is always difficult. So far, the one test that successfully separated the bet‐hedging effect from the sexual selection process was carried out by an artificial insemination experiment in a sea urchin with external fertilization (Garcia‐Gonzalez et al., 2015). Although our study using a species with internal fertilization could not separate bet‐hedging and sexual selection, it does represent a novel empirical attempt to test the model. To comprehensively test the bet‐hedging polyandry hypothesis, additional empirical studies using various species are needed. In addition, our statistical test of geometric mean fitness (Figure 4) highlights the problems of insufficient statistical power in randomization tests. Statistical methods for treating the geometric mean should be further developed.

## CONFLICT OF INTEREST

The authors declare no conflict of interest.

## AUTHOR CONTRIBUTION

**Kentarou Matsumura:** Conceptualization (equal); Data curation (equal); Formal analysis (equal); Funding acquisition (equal); Investigation (equal); Methodology (equal); Project administration (equal); Resources (equal); Software (equal); Supervision (equal); Validation (equal); Visualization (equal); Writing‐original draft (equal); Writing‐review & editing (equal). **Takahisa Miyatake:** Conceptualization (equal); Funding acquisition (equal); Investigation (equal); Methodology (equal); Project administration (equal); Resources (equal); Supervision (equal); Validation (equal); Visualization (equal); Writing‐original draft (equal); Writing‐review & editing (equal). **Yukio Yasui:** Conceptualization (equal); Formal analysis (equal); Funding acquisition (equal); Investigation (equal); Methodology (equal); Project administration (equal); Resources (equal); Software (equal); Supervision (equal); Validation (equal); Visualization (equal); Writing‐original draft (equal); Writing‐review & editing (equal).

## Supporting information

Supplementary Material

## Data Availability

The data associated with this publication can be accessed on Dryad (https://doi.org/10.5061/dryad.2rbnzs7n2).
