# Supplementary material for: An empirical test of the bet‐hedging polyandry hypothesis: Female red flour beetles avoid extinction via multiple mating
Source: Ecol Evol. 2021 Mar 18;11(10):5295–304. doi: 10.1002/ece3.7418 (PMC8131809; doi:10.1002/ece3.7418)
Supplement: Supplementary file 1 — Supplementary Material [file ECE3-11-5295-s001.docx]

Supplementally material

**Table S1**. AIC values under various frequency distribution regarding the number of eggs and larvae in generalized liner model (GLM).

| Dependent variable | Distribution | AIC value |
| --- | --- | --- |
| Number of eggs | Gaussian | 606.84 |
|  | Poisson | 1166.4 |
|  | Gamma | 603.72 |
| Number of larvae | Gaussian | 596.48 |
|  | Poisson | 1690.3 |
|  | Gamma* | 612.96 |

*Because the gamma distribution is only for positive values, we added 1 to the data.
